# A potential role of a special type of abortive seeds in *Cunninghamia lanceolata*: promoting the growth of healthy seedlings in active aluminum ions-rich soil

**DOI:** 10.3389/fpls.2024.1482355

**Published:** 2024-11-08

**Authors:** Shi-Yan Mu, Ya-Ting Yang, Xiao-Yu Qu, Fang-Fang Wang, Fang-Fang Ma, Zhen-Ning Ding, Ling-Peng Ye, Ya-Ling Zhang, Jia-Jun Zhang, Meng-Meng Lyu, Shu-Bin Li, Guang-Qiu Cao, Chao Wu, Guo-Chang Ding, Yu Chen

**Affiliations:** ^1^ College of Forestry, Fujian Agriculture and Forestry University, Fuzhou, China; ^2^ Key Laboratory for Forest Stress Physiological Ecology and Molecular Biology of Fujian Provincial Department of Education at College of Forestry, Fujian Agriculture and Forestry University, Fuzhou, China; ^3^ Chinese Fir Engineering Technology Research Center of the State Forestry and Grassland Administration at College of Forestry, Fujian Agriculture and Forestry University, Fuzhou, China; ^4^ College of Computer and Information Science, Fujian Agriculture and Forestry University, Fuzhou, China

**Keywords:** *Cunninghamia lanceolata*, astringent seeds, aluminum toxicity, alleviative effect, seedling growth, soil microenvironment mediation

## Abstract

**Background and aims:**

“Astringent seed” is a type of abortive seed frequently observed in Chinese fir (*Cunninghamia lanceolata*). It is widely recognized but poorly understood for its underlying causes. This study investigates the potential of astringent seeds to alleviate the toxic effects of active aluminum ions.

**Methods:**

This study involved treating seeds and seedlings with two distinct concentrations of astringent seeds water extracts under the aluminum ion stress. Then the germination of seeds and growth of seedlings were evaluated and compared.

**Results:**

Under aluminum stress, both seed germination and seedling growth were notably inhibited. Treatment with a low-concentration of the extract significantly alleviated this inhibition. Root elongation in the seedlings increased by 36.95% compared to the control group, and the aluminum ion accumulation at the root tips was reduced by 38.89% relative to the aluminum-stressed group. This treatment also normalized the levels of malondialdehyde (MDA) in the roots and leaves, enhanced the activities of antioxidative enzymes such as superoxide dismutase (SOD) and catalase (CAT), and restored the levels of endogenous hormones including gibberellin (GA_3_), indole-3-acetic acid (IAA), methyl jasmonate (Ja-ME), and abscisic acid (ABA). Furthermore, the low-concentration of the extract positively impacted the disorganized chloroplast structures. In contrast, a high-concentration of the extract failed to revert most of these stress indicators.

**Conclusion:**

Low concentrations of astringent seed water extract effectively alleviate the inhibitory effects of aluminum ions on seed and seedling. This implies that in natural environments, the proximity of healthy seeds to astringent seeds could potentially enhance their growth.

## 1 Introduction

Seed abortion is a widespread occurrence in plants, influenced by various factors such as developmental abnormalities in organs or tissues (e.g., pollen grains, stigmas, ovaries, female gametophytes) (Li et al., 2009, 2021.,Cardel and Koptur, 2022), self-incompatibility, zygote development issues post-fertilization, and the presence of homozygous lethal genes (Xie et al., 2019; Shen et al., 2020; Wang et al., 2023). Resource allocation and elimination are often reflected in abortive seeds, with the vast majority of abortive seed embryos ultimately degenerating and disappearing or being significantly smaller than the embryos of viable seeds (Chen et al., 2022; Wang et al., 2023). Consequently, healthy seeds are allocated more nutrients, ensuring their subsequent germination and growth.

**Figure 1:**
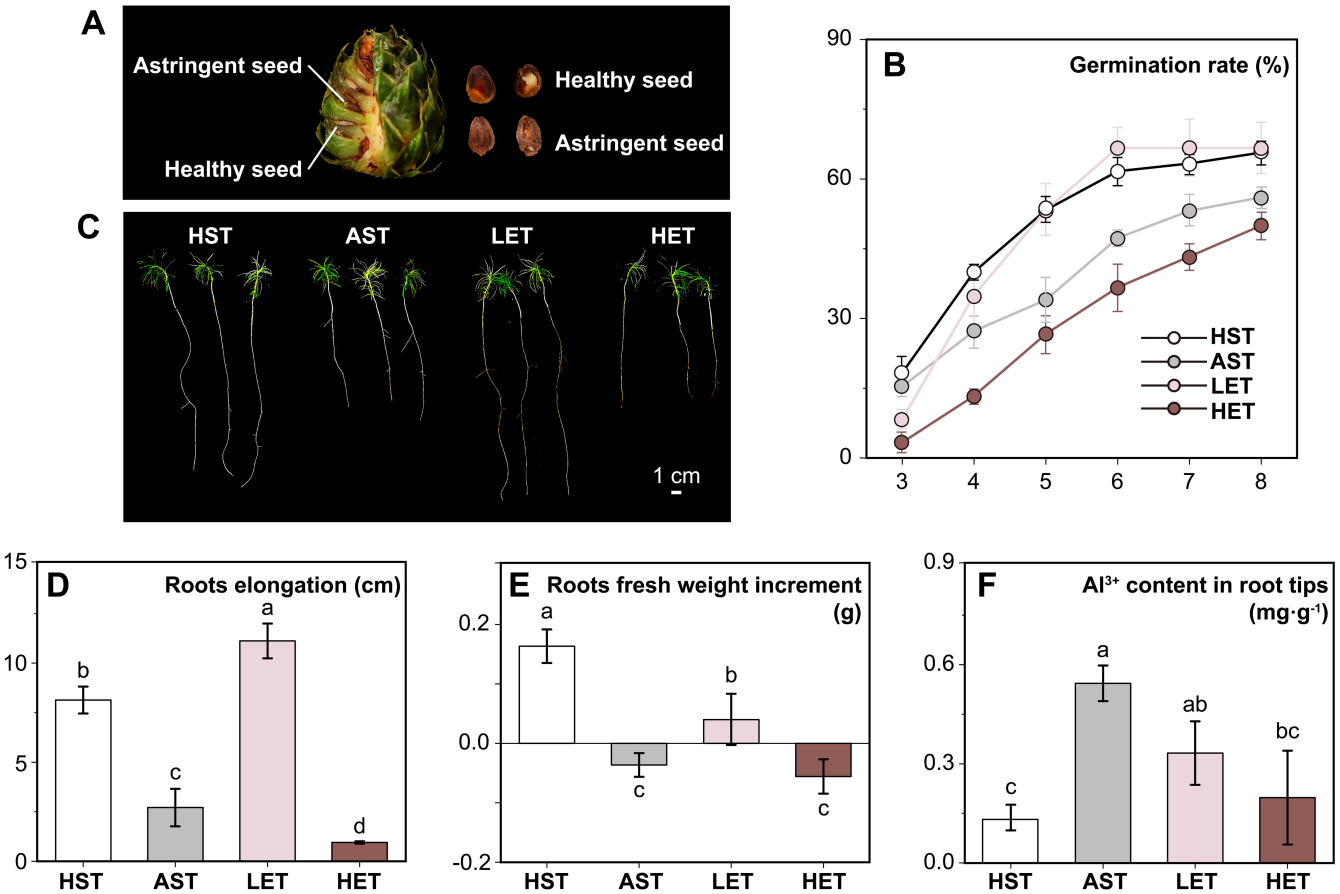
Effects of astringent seeds extract treatments on seed germination and seedling root growth under aluminum ions stress. The astringent seeds used in this study, although appearing normal, are an aborted type **(A)**. Compared to the HST, the germination rate of AST significantly decreased. This decrease was alleviated by low-concentration extract treatment, but high-concentration extract treatment did not restore the germination rate **(B)**. Seedling growth under different treatments showed varied performances **(C)**. Root elongation **(D)** and the increase in root fresh weight **(E)** were significantly inhibited in the AST group. However, in LET group, seedling roots elongated noticeably, and the inhibition of fresh weight increase was significantly reduced. Under the aluminum stress treatment, the Al^3+^ ion content in seedling root tips increased significantly. This increase was significantly alleviated by low-concentration extract treatment and to a lesser extent by high-concentration extract treatment **(F)**. The results of multiple comparisons are indicated above the bar chart. Treatments marked with the same letter have no significant difference (p > 0.05).

However, in Chinese fir (*Cunninghamia lanceolata*), there is a unique type of aborted seed, which is specifically named the astringent seed. These astringent seeds are indistinguishable from healthy seeds by simple physical methods, as they appear identical in appearance (**Figure 1A**) and have similar weights. In essence, these astringent seeds seem to “grow” even though their embryos are dead (Zheng et al., 2023). The true nature of these seeds is only revealed when the seed coat is removed, exposing their deep red or dark brown color, which contrasts sharply with the milky white or light yellow of healthy seeds. Unfortunately, astringent seeds are prevalent in high-quality seed orchards of *C. lanceolata*. In some orchards, the rate of astringent seeds in superior families has approached 90% (Lin et al., 2022). This high incidence of astringent seeds significantly reduces both the yield and quality of improved seeds. Additionally, their resemblance to healthy seeds complicates their identification, leading to increased resource wastage during seedling cultivation (Zheng et al., 2023). In exploring this phenomenon, a key question emerges naturally: what is the benefit of such an energy-intensive “investment”? We hypothesize that, in nature, the distribution of astringent seeds of the *C. lanceolata* after maturation and detachment from the parent plant should be random. This randomness suggests that astringent seeds often fall near healthy ones. Therefore, we propose that astringent seeds may play a supportive role for viable seeds, as they likely coexist in close proximity, collectively enduring adverse environmental conditions.

Coincidentally, in southern China, the primary distribution area of the extant *C. lanceolata* (Jiang et al., 2022), the soil type is acidic red soil (Shen et al., 2021), which is characterized by relatively high levels of active aluminum ions (Zheng and Li, 2004). Luo et al. (2014) revealed that the seasonal variation of active aluminum ions in different soil layers of *C. lanceolata* plantations ranges approximately from 1.98 to 3.04 mg·g^-1^, indicating that this important timber species is also subject to the toxic effects of active aluminum ions. The harmful impact of active aluminum ions has been well-documented, with studies consistently demonstrating that the most noticeable morphological change under aluminum ion stress is the inhibition of root tip elongation (Bai et al., 2017; Muhammad et al., 2018; Yang et al., 2019., Sun et al., 2020). For instance, in maize, the region 1-2 mm from the root tip is particularly sensitive to aluminum ions, resulting in inhibited cell division in the meristematic tissue of this area (Eticha et al., 2005). Additionally, plants under aluminum ion stress exhibit physiological responses such as reduced photosynthesis and respiration, disruption of redox balance, and imbalances in endogenous hormone metabolism (Cárcamo et al., 2019). Interestingly, it has been found that astringent seeds are rich in flavonoids/polyphenolic secondary metabolites and their derivatives (Chen et al., 2020), which are the basis for their name. These secondary metabolites have been shown to possess the ability to detoxify active aluminum ions (Chen and Wang, 2003; Chen et al., 2020; Zheng et al., 2023), potentially offering a protective mechanism against the toxic effects observed in plants.

Therefore, we further hypothesize that astringent seeds may play a role in reducing active aluminum content in the soil, thereby assisting the growth of seedlings with lower resistance to this toxin. To investigate this, we treated aluminum-damaged *C. lanceolata* seeds and seedlings with water extracts of astringent seeds and monitored their growth and physiological response change. This approach aims to provide a preliminary explanation for the aforementioned hypothesis and to offer a new perspective and foundational basis for future in-depth studies.

## 2 Materials and methods

### 2.1 Preparation of astringent seeds water extracts

Based on previous research findings, we prepared two concentrations of astringent seeds water extracts from *C. lanceolata* seeds: 1.00 g·L^-1^ and 100 g·L^-1^. Initially, the appropriate weight of dried seeds was measured and ground into a fine powder. This powder was then immersed in distilled water and shaken for 48 hours to ensure complete dissolution of the soluble substances. The solution was subsequently filtered, and the residue was thoroughly squeezed to minimize liquid loss. These water extracts of varying concentrations will be used as solvents in subsequent experiments, replacing distilled water.

### 2.2 Seedling cultivation

Seeds of *C. lanceolata* were purchased from the third-generation seed orchard of Youxi National Forest Farm in Fujian Province, China. The seedlings were cultured in Hoagland nutrient solution in a hydroponic system for 15 days after germination. Seedlings with similar height and root length were then selected for further tests.

### 2.3 Aluminum stress and astringent seed water extraction treatment

The selected seedlings were transferred to new hydroponic systems. Seedlings cultivated in pure Hoagland solution treatment (HST) served as the control group. To create the active aluminum stress treatment (AST) group, a solution of 0.5 mmol·L^-1^ AlCl_3_·6H_2_O was added into the Hoagland nutrient solution. Based on the AST group, two concentrations of astringent seeds water extracts were incorporated, forming the low-concentration extract treatment (LET) and high-concentration extract treatment (HET) groups. Each of the 4 groups had three replicates, with each replicate containing 30 seedlings. The *p*H of the solutions in all groups was adjusted to 4.5 and readjusted every 3 days throughout the 30-day cultivation period.

### 2.4 Determination of seedling growth

Before starting the stress and extract treatments experiments, the total plant length, root length, and fresh weight of all seedlings were measured. Additionally, 30 seedlings that were not part of the experiments were divided into root and shoot sections, and their fresh weights were recorded separately. These initial measurements provided the baseline averages for subsequent weight increment calculations. Following the active aluminum stress and extract treatments, the total plant length, root length, and fresh weights of the whole plant, roots, and shoots were re-measured. The growth increments for each seedling under different treatments were determined by comparing the initial and final measurements.

### 2.5 Determination of antioxidant enzyme activity in seedlings

The content of malondialdehyde (MDA), superoxide dismutase (SOD), catalase (CAT), and peroxidase (POD) in seedlings were investigated to assess antioxidant levels. MDA content was determined by the thiobarbituric acid (TBA) colorimetric method (Morales and Munné-Bosch, 2019), SOD activity by the nitrotetrazolium blue chloride (NBT) photochemical reduction method (Zahir et al., 2021), CAT activity by the UV absorption method (Gordo et al., 2024), and POD activity by the guaiacol test process (Mika and Lüthje, 2003).

### 2.6 Determination of endogenous hormones in seedlings

The contents of endogenous hormones, including gibberellin (GA_3_), indoleacetic acid (IAA), zeatin (ZT), methyl jasmonate (Ja-ME), and abscisic acid (ABA), were determined using an enzyme-linked immunosorbent assay (ELISA) kit (SINOVAC Biotechnology Co., Ltd., Shanghai, China), and the assay was performed according to the manufacturer’s instructions.

### 2.7 Determination of chlorophyll content in seedlings

After aluminum stress and water extract treatments, leaves from *C. lanceolata* seedlings under each treatment were cut (the 4th to 6th leaves from the top of the seedlings). The contents of chlorophyll a (Chla), chlorophyll b (Chlb), total chlorophyll, and carotenoid were determined using the extraction method (Berhe et al., 2024).

### 2.8 Observation of organelle structure in seedling leaves

The leaf samples used were the same as those selected for the determination of chlorophyll content described in section 2.7. They were fixed with 0.2 mol·L^-1^ phosphate buffer and 25% glutaraldehyde fixative solution, then dehydrated, embedded, cured, sliced, and observed using an Tecnai Spirit G2 Bio TWIN transmission electron microscope (FEI, Hillsboro, USA).

### 2.9 Data processing

Multiple comparisons were performed to determine significant differences in performance between groups using the Duncan test, conducted with SPSS v27 software. Origin v2022 software was used to visualize the data.

## 3 Results

### 3.1 Effects of extract treatments on germination of seeds

Germination commenced on the third day across all experimental groups (**Figure 1B**). The HST and AST groups displayed relatively higher initial germination rates of 18.33% and 15.36%, respectively, followed by the LET group (8.16%). The HET group exhibited the lowest germination rate at 3.33%. From the third to the sixth day, germination rates in the HST, AST, and LET groups increased rapidly but slowed thereafter. By the eighth day, the HST group, serving as the control, reached a germination rate of 65.31%. The AST group was lower significantly (*p* < 0.05) at 55.61%, and the LET group achieved a rate comparable to the HST at 66.45%. In contrast, the HET group’s germination rate remained significantly lower throughout the observation, not only compared to the control group, but also to the AST group. Notably, the peak germination rate for the HET group had not occurred by the eighth day, indicating a delayed response compared to the other treatments. In conclusion, the data suggest that aluminum stress adversely affects the vigor and germination of *C. lanceolata* seeds. Low-concentrations extract treatment appeared to alleviate some of this stress, whereas high-concentrations not only failed to provide relief but further intensified the inhibition.

### 3.2 Effects of extract treatments on seedling roots in active aluminium stress

The growth of *C. lanceolata* seedlings’ root systems under active aluminum stress and varying concentrations of astringent seeds water extracts showed significant differences (**Figure 1C**). Compared to the HST group, the AST group experienced a 66.63% reduction in root elongation (**Figure 1D**) and a 112.50% decrease in fresh weight increment (**Figure 1E**). This inhibition was notably mitigated in the LET group, where root elongation was 36.95% greater than in the HST group. However, the fresh weight increment in the LET group was significantly lower (*p* < 0.05) than that in the HST group, though still higher than in the AST group. This indicates that low concentrations of astringent seeds water extracts can enhance root elongation, but may still impact cellular density within root tissues. In the HET group, both root elongation and fresh weight increment were significantly lower than those in the HST group. Additionally, the Al^3+^ ions content in the root tips of the AST group was 0.54 mg·g^-1^. In comparison, the LET and HET groups showed reductions of 38.89% and 62.96%, respectively (**Figure 1F**), indicating that astringent seeds water extracts can inhibit the absorption of Al^3+^ ions by the roots. However, excessively high concentrations, while reducing the Al^3+^ content in the root tips, also have inhibitory effects through other potential pathways.

**Figure 2:**
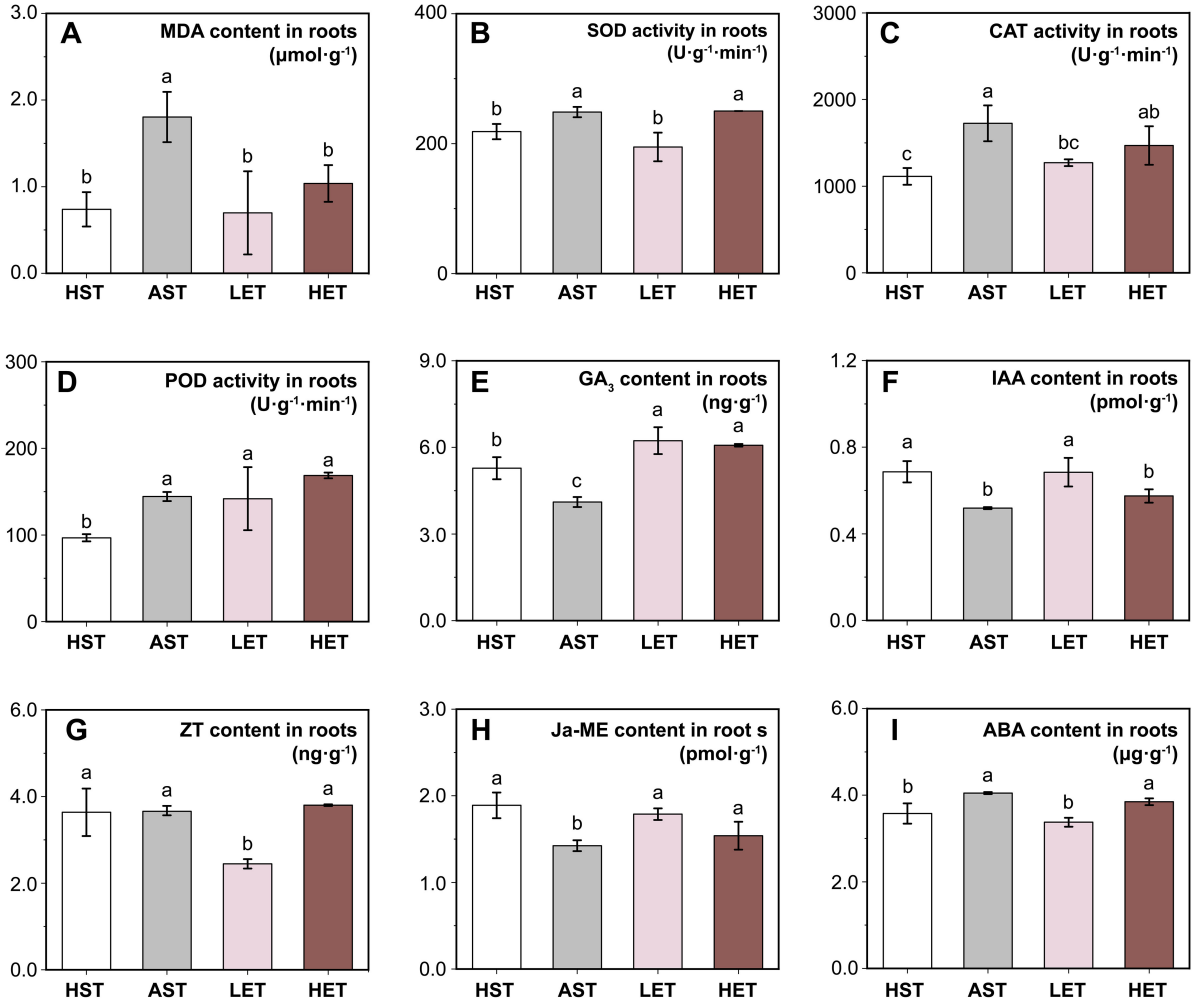
Effects of different treatments on antioxidant enzyme activities and endogenous hormone levels in roots. Four antioxidant-related indicators: MDA content **(A)**, SOD activity **(B)**, CAT activity **(C)**, and POD activity **(D)**, and levels of five endogenous hormones: GA_3_ **(E)**, IAA **(F)**, ZT **(G)**, Ja-ME **(H)**, and ABA **(I)** are presented. Most physiological parameters approached normal, unstressed levels under the LEG treatment, while the HEG treatment failed to produce similar outcomes. The results of multiple comparisons are indicated above the bar chart. Treatments marked with the same letter have no significant difference (p > 0.05).

The MDA content in the root systems of the AST group increased by 58.89% compared to the HST group (**Figure 2A**), indicating increased membrane permeability in root tissues under aluminum stress. Additionally, the activities of other antioxidant enzymes showed significant increases (**Figures 2B-D**), suggesting the activation of physiological stress responses in the tissues. The LET group demonstrated a noticeable recovery effect, with MDA content, SOD, and CAT activities returning to levels similar to those of the HST group. In the HET group, a significant reduction in MDA content was observed, although the activities of the three antioxidant enzymes remained significantly higher (*p* < 0.05) than in the HST group.

The endogenous GA_3_ (**Figure 2E**) and IAA (**Figure 2F**) levels in seedling roots showed similar trends. Both decreased significantly (*p* < 0.05) under aluminum stress and increased following treatment with extracts of astringent seeds, regardless of concentration. Notably, the GA_3_ content in the HET group was significantly higher than in the HST group, suggesting that high concentrations of extract might mitigate the inhibitory effect of aluminum stress on the biosynthesis of GA_3_. However, the IAA content did not return to normal levels. The endogenous ZT content (**Figure 2G**) followed a different pattern. There were no significant changes in ZT content in the root systems under aluminum stress, while a significant decrease was observed in the LET group. Changes in Ja-ME content (**Figure 2H**), which is related to stress response, mirrored those of GA_3_ and IAA. The ABA content in the AST group significantly increased (*p* < 0.05) but returned to levels comparable to the HST group in the LET group and showed no significant difference from the ATG group in the HEG group (**Figure 2I**).

### 3.3 Effects of extract treatments on seedling leaves in active aluminium stress

**Figure 3:**
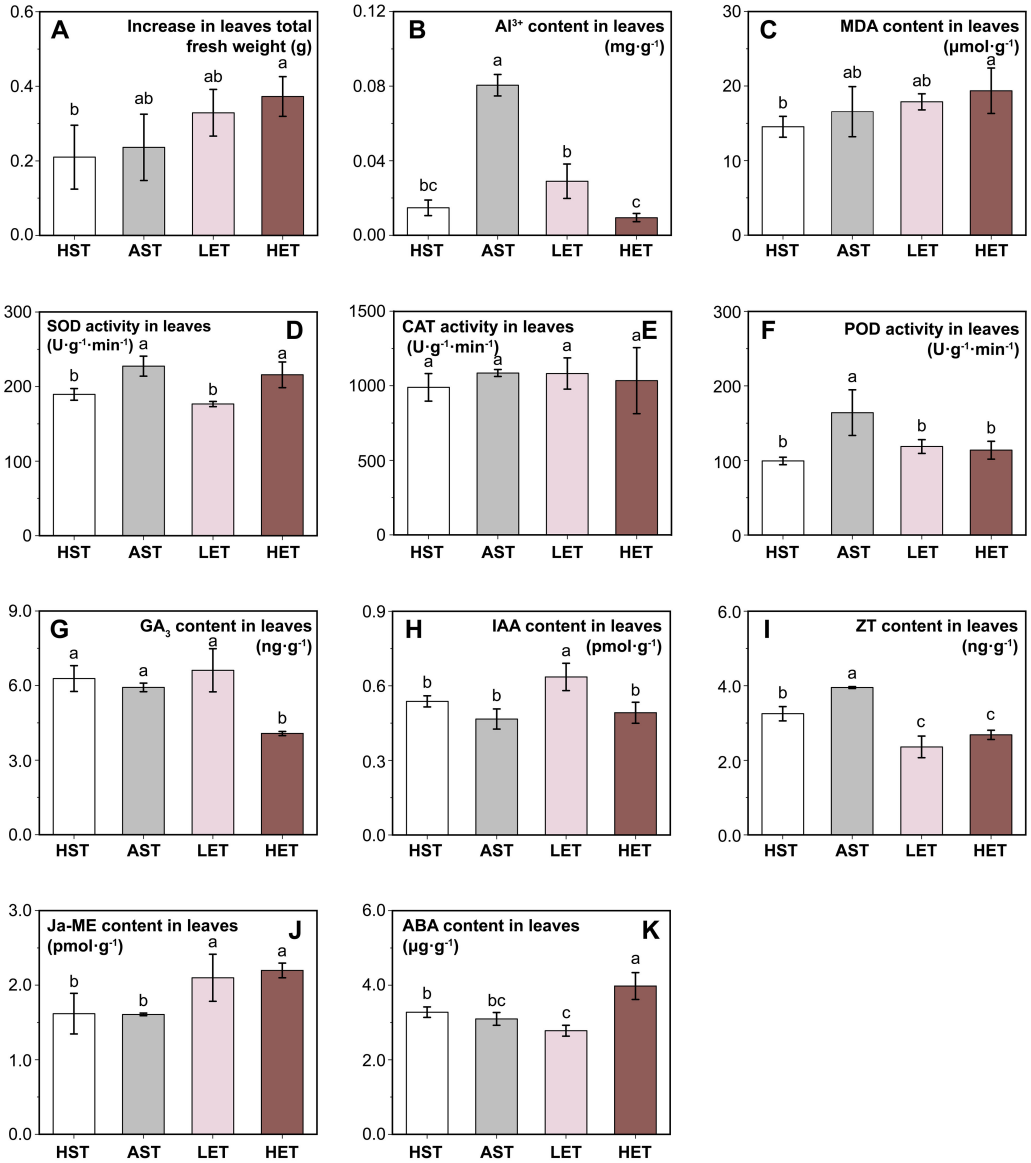
Effects of different treatments on seedling leaves. Leaves fresh weight increment **(A)**, Al^3+^content **(B)**, four antioxidant-related indicators: MDA content **(C)**, SOD activity **(D)**, CAT activity **(E)**, and POD activity **(F)**, and levels of five endogenous hormones: GA_3_ **(G)**, IAA **(H)**, ZT **(I)**, Ja-ME **(J)**, and ABA **(K)** in leaves are presented. The results of multiple comparisons are indicated above the bar chart. Treatments marked with the same letter have no significant difference (p > 0.05).

Aluminum stress does not seem to affect the leaf growth of *C. lanceolata* seedlings visibly. The increase in leaf fresh weight in the AST group showed no significant difference compared to the HST group (**Figure 3A**), and the same was recorded for the LET group. However, the HET group exhibited a 76.19% higher increase in leaf fresh weight increment compared to the HST group. In contrast, significant differences were observed in the active Al^3+^ content in the leaves (**Figure 3B**). The active Al^3+^ content in the AST group reached 0.08 mg·g^-1^, but it decreased by 28.40% and 45.68% in the LET and HET groups, respectively. This suggests that the extract may influence aluminum ions absorption and transport.

The MDA content in the leaves of the AST group did not show a significant difference compared to the HST group. However, in the LET group was 33.25% higher than in the HST group (**Figure 3C**). The activities of the three antioxidant enzymes exhibited varying patterns. Leaf SOD activity significantly increased under aluminum stress and remained at a similar level in the LET group as in the HST group but did not decrease in the HET group (**Figure 3D**). No significant differences in CAT activity were observed among the four groups (**Figure 3E**). POD activity showed a significant increase (*p* < 0.05) only in the AST group, while the activities in the other three experimental groups were nearly identical (**Figure 3F**).

The endogenous hormone levels in the leaves also exhibited different patterns under various treatments. The levels of GA_3_ (**Figure 3G**) and IAA (**Figure 3H**) contents were significantly different in the HET and LET groups, but both were unaffected by aluminum stress. The endogenous ZT content in the AST group was significantly higher than in the HST group, while in the LET and HET groups were significantly lower than those in the HST group (**Figure 3I**). Compared to the HST group, the Ja-ME content in the leaves showed no significant change in the AST group but increased markedly in both the LET and HET groups (**Figure 3J**). The ABA content also significantly decreased in the LET group and increased in the HET group (**Figure 3K**). This suggests that aluminum stress has a minimal impact on endogenous hormone synthesis in leaves, whereas the astringent seeds extract introduces a new regulatory factor affecting hormone levels.

**Figure 4:**
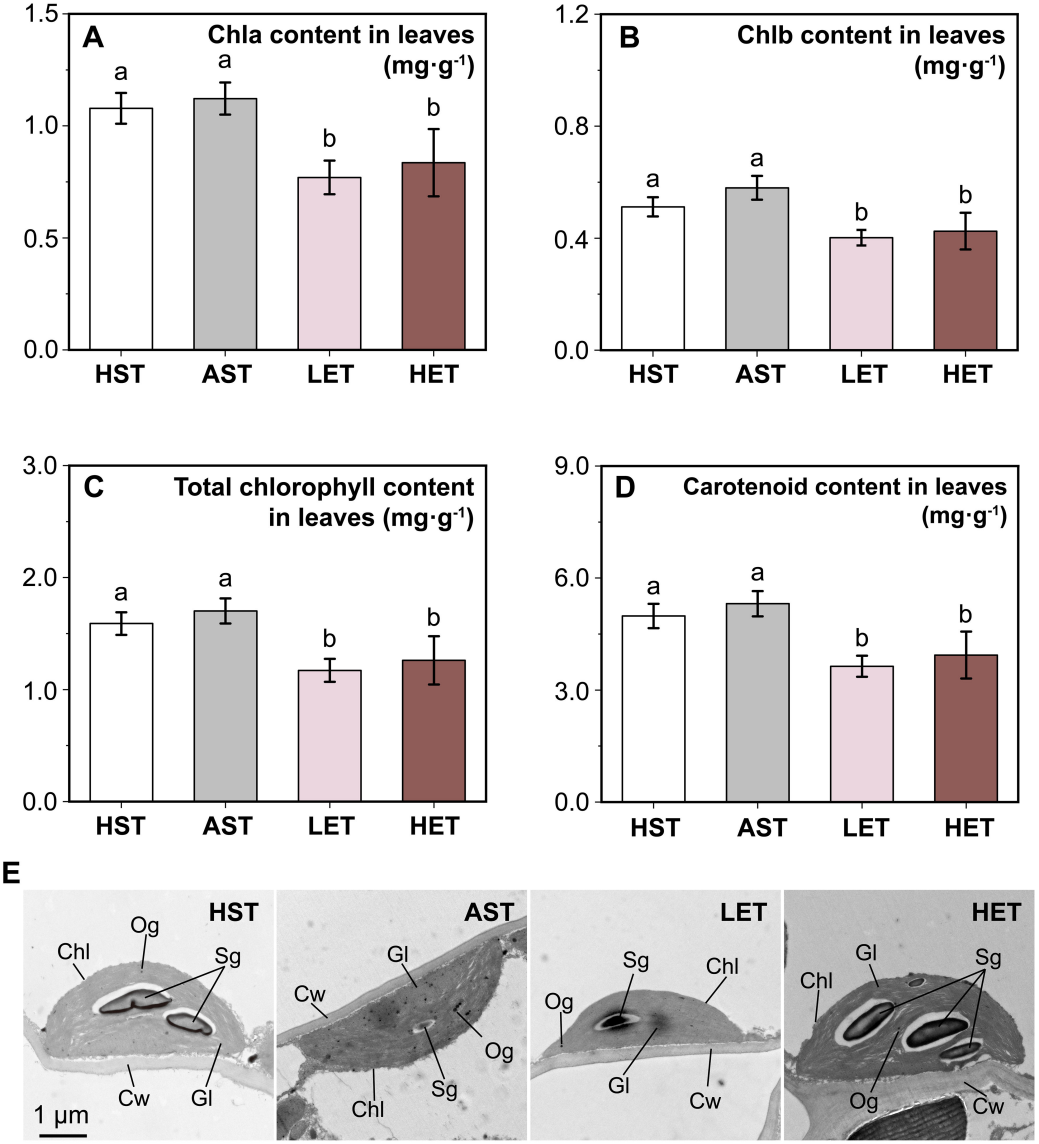
Impact of different treatments on chlorophyll content and chloroplast organelle structure. Changes in photosynthetic pigments, including chlorophyll a **(A)**, chlorophyll b **(B)**, total chlorophyll **(C)**, and carotenoids **(D)**, following different treatments are displayed. Additionally, the ultrastructure of chloroplasts **(E)** is shown, highlighting differences in chloroplasts (Chl), cell walls (Cw), starch grains (Sg), grana lamella (Gl), and osmiophilic globules (Og) across various treatments. Notably, the LEG treatment helped to reduce the disorganization of chloroplast structures induced by aluminum stress. The results of multiple comparisons are indicated above the bar chart. Treatments marked with the same letter have no significant difference (p > 0.05).

The chlorophyll content in leaves followed a similar pattern across different treatments. There were no significant differences in chlorophyll a (**Figure 4A**), chlorophyll b (**Figure 4B**), total chlorophyll (**Figure 4C**), and carotenoid contents (**Figure 4D**) between the HST and AST groups. However, under the influence of astringent seeds extract treated, the contents of these photosynthetic pigments significantly decreased (*p* < 0.05). Transmission electron microscopy was used to observe the organelle structure changes of the chloroplast under various treatments. In the HST group, chloroplast structures were intact (**Figure 4E**). They were closely attached to the cell wall, spindle-shaped, with clear and tightly arranged grana lamellae, noticeable gaps between starch grains and thylakoids, and a high number of osmiophilic granules. In the AST group, cell walls appeared wrinkled and curved, with a reduced number of osmiophilic granules, blurred starch grains, and loosely arranged grana lamellae. This indicates that while chlorophyll content did not significantly decrease in the aluminum-rich environment, the structure was still affected by stress. In the LET group, chloroplast starch grains were clear, and grana lamellae were tightly arranged, but the number of osmiophilic granules remained very low. This suggests that low concentrations of extract positively affect the loose chloroplast structure caused by aluminum stress, although the improvement is incomplete. In the HET group, the volume of starch grains increased, osmiophilic granules appeared, and grana lamellae were relatively loosely arranged.

## 4 Discussion

### 4.1 Mechanism of plant response to active aluminum ion toxicity

Aluminum is one of the most abundant elements in the Earth’s crust, surpassed only by oxygen and silicon. In neutral or alkaline soils, aluminum predominantly exists as insoluble aluminum silicates or aluminum oxides, posing no harm to plants (Eticha et al., 2005; Zheng, 2010; Wei et al., 2020). However, in acidic soils, aluminum ions dissolve and form various active valence states, such as Al^3+^, Al(OH)^2+^, Al(OH)_2_^+^, Al(OH)_3_, and aluminum-humic acid complexes (Al-HA) (Zhou et al., 2011). These active forms of aluminum are toxic to plants (Shetty et al., 2020), linking aluminum ion toxicity closely with soil acidification (Drabek et al., 2003).

Plants living in acidic soil have developed mechanisms to cope with the challenging conditions posed by active aluminum ions. These adaptation mechanisms are broadly categorized into two types: external exclusion and internal tolerance, distinguished by the location of detoxification within the plant (Kochian et al., 2015; Sade et al., 2016). External exclusion primarily occurs in the apoplast, where organic acids from the cytoplasm bind to aluminum, and vacuoles help isolate these complexes (Yan et al., 2023). This process transforms harmful aluminum ions into harmless or less harmful forms, mitigating their toxic effects. Internal tolerance, on the other hand, takes place in the symplast (Yang et al., 2019). Here, root tips release organic acids, phosphates, phenolic compounds, or mucilage that bind with aluminum ions (Yang et al., 2013, 2019). Root exudates also help adjust the *p*H around the root zone, creating a barrier that lowers the likelihood of aluminum ions penetrating root cell tissues (Ma and Lin, 2019). However, it’s important to note that these physiological responses are typically more developed in mature plants (Vicente et al., 2020; Parkash et al., 2024). Seeds and seedlings may not fully express these protective mechanisms, making them more vulnerable to aluminum toxicity.

### 4.2 Alleviating effect of astringent seed on aluminum toxicity

As previously mentioned, the soil in the distribution area of *C. lanceolata* contains relatively high levels of active aluminum ions, posing a potential toxic threat to the species. In response, it has developed adaptive mechanisms to withstand this environmental stress. Research by Ma and Lin (2019) shows that aluminum stress triggers the biosynthesis of polyphenolic compounds in *C. lanceolata* roots, along with notable changes in antioxidant activity. Our own observations confirm that aluminum stress enhances antioxidant responses in the roots of seedlings. However, given that seedlings have underdeveloped root systems, they may require additional mechanisms to manage such challenging conditions. Astringent seeds could play a critical role in this adaptation.

**Figure 5:**
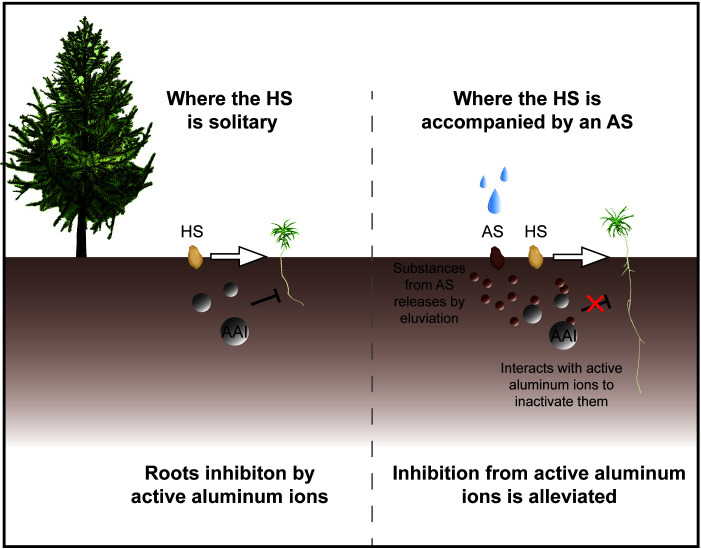
Potential mechanisms of astringent seeds in mitigating toxicity of active aluminum ions in soil. The protective role of astringent seeds (AS) in natural environments is illustrated. In conditions where solitary healthy seeds (HS) are exposed to soil with active aluminum ions (AAIs), their germination and seedling growth are typically hindered. However, the presence of astringent seeds can alter this outcome. Substances leached from tannin seeds by eluviation permeate the surrounding soil, effectively inactivate the toxic impact of active aluminum ions and thereby safeguarding the germination and growth of nearby healthy seeds and seedlings.

While our results focus mainly on physiological changes, they do support the conclusion that astringent seeds can mitigate the effects of active aluminum stress on healthy seeds. This finding suggests that the astringent seeds of *C. lanceolata* may have evolved as a specialized form of abortive seeds, adapted to thrive in environments with high levels of active aluminum (**Figure 5**). Astringent seeds, when they fall into the soil along with healthy seeds, may release their substance into the surrounding soil by the eluviation. This can potentially regulate the local concentration of active aluminum ions. Predominantly composed of flavonoids and polyphenolic compounds in the astringent seeds (Lin et al., 2017; Chen et al., 2020) are known to bind with active aluminum ions by forming chelates (Ma and Lin, 2019). Moreover, this possible adaptive mechanism appears particularly advantageous for the growth of seedling roots, which are highly susceptible to aluminum ion toxicity (Ofoe et al., 2022). When present in optimal concentrations, this mechanism not only promotes root growth but also helps restore most physiological responses related to resistance and endogenous hormone levels to their normal state before stress (**Figures 1**, **2**). For instance, the notable reduction in ABA content in the roots of the LET group suggests this recovery effect. Conversely, this recovery effect is less pronounced in the leaves, where a reduction in photosynthetic pigment content is observed. Nonetheless, changes in chloroplast structure in the leaves provide further evidence of adaptation (Venzhik et al., 2019). Morphological adaptations in chloroplasts, such as the arrangement of granal lamellae observed in the LET group, indicate a mitigated impact of active aluminum, demonstrating the leaves’ ability to adjust to environmental stresses.

Our results also highlight the importance of concentration in determining the impact of water extract on *C. lanceolata* seedlings. High-concentration treatments inhibited seedling growth, affecting both root development (**Figures 2**, **3**) and leaf physiology (**Figures 4**, **5**). Notably, these inhibitory effects occurred even in the absence of aluminum ions (**Supplementary Figure S1**), suggesting that the high-concentration extract itself may be a growth inhibitor. This aligns with the typical concentration-dependent behavior of allelochemicals, which can promote growth at low concentrations but become inhibitory at higher levels (Kato-Noguchi et al., 1994; Soln et al., 2022; Kumar et al., 2024). However, the potential interaction between the extract and active aluminum ions remains uncertain. One hypothesis is that high concentrations of the extract might reduce the toxicity of active aluminum ions while simultaneously exerting its own negative impact on seedling growth. An alternative explanation is that the extract does not mitigate aluminum toxicity, and the observed growth inhibition is due to the combined effects of both stressors. Further investigation is required to determine the nature of these interactions.

Certainly, to fully understand how *C. lanceolata* seedlings cope with the toxic effects of active aluminum ions and the mechanism by which the secondary metabolites in astringent seeds interact with active aluminum, further research is required. For example, a comparative analysis of organic acid exudation between the roots of mature trees and seedlings could reveal whether *C. lanceolata* primarily employs one of the two proposed strategies to mitigate aluminum toxicity and whether seedlings exhibit weaker resistance to this stress. Additionally, investigating changes in the form and concentration of active aluminum ions in the soil before and after treatment with astringent seed extracts could determine if these extracts effectively reduce aluminum toxicity. Our current findings provide physiological insights that may serve as a foundation for these more detailed studies.

### 4.3 The significance of astringent seeds and their potential benefits to healthy seeds

Seed abortive phenomenon is understood through the lens of plant-environment interactions and adaptation, shaped by resource limitations (Burd, 1998) and adaptive evolution (Meyer et al., 2014). Producing seeds is an energy-intensive process, offering plants the chance to disperse their genetic material and ensure the continuation of their species (Lee et al., 2016; Nguyen et al., 2016; Jayakodi et al., 2019; Du et al., 2024). However, the availability of resources, which depends on the plant’s nutrient accumulation and recycling rates, often restricts the simultaneous survival of all seeds (Godoy et al., 2016). Therefore, nutrient allocation among seeds of different genotypes is critical. Over long-term evolutionary processes, plants have developed the ability to prioritize resource investment in more advantageous genotypes while sacrificing weaker ones (Aastveit and Aastveit, 1993). External factors such as insect infestation, physical damage, and environmental stress can also trigger this elimination mechanism, sometimes overshadowing the influence of genotype superiority (Plomin and Asbury, 2005; Arathi, 2011; Orgogozo et al., 2015). Therefore, seed abortion is viewed as having significant evolutionary importance.

Our findings suggest that astringent seeds may assist seedlings in managing the stress from active aluminum ions in acidic red soils. However, the possibility of astringent seeds serving additional roles merits further investigation. One potential role could be the alteration of the soil microenvironment within a localized area, potentially giving *C. lanceolata* seedlings a competitive edge by adversely affecting other plants. Plants are known to release specific metabolites through external secretion or during litter decomposition, influencing neighboring vegetation either directly or indirectly (Chomel et al., 2016; Yang et al., 2021; Schroeter et al., 2022). In this context, our observations indicated a delay in the germination of *C. lanceolata* seeds in the presence of HST group (**Figure 1A**). This delay may not just be an evasion of the toxic effects of these high concentrations but could also signify an adaptive strategy. As rainfall dilutes these extracts, seeds might find an enhanced germination opportunity once competing species have been diminished. Another hypothesis is that astringent seeds may deter consumption by wildlife, including insects, birds, and rodents. Studies indicate that seeds with higher tannin content are less appealing to these animals (Zhang et al., 2013, 2024). Given that astringent seeds are aborted seeds with elevated tannin levels, it is plausible that their presence among healthy seeds could deter potential feeders, thereby reducing predation rates.

Both hypotheses, however, require additional empirical evidence to be substantiated.

## 5 Conclusions

This study investigates whether astringent seeds of *C. lanceolata* enhance the germination and seedling growth by mitigating the effects of active aluminum ion stress. Healthy seeds and seedlings treated with different concentrations of astringent seed water extracts were evaluated through comparative analyses of germination rates, seedling growth, antioxidant physiology, and endogenous hormone levels. The findings suggest that low concentrations of these extracts can help shield healthy seeds from active aluminum ion stress, facilitating a partial recovery of their normal physiological functions.

## Data Availability

The original contributions presented in the study are included in the article/**Supplementary Material**. Further inquiries can be directed to the corresponding author.

## Supplementary material

Supplementary Figure 1 Differences in root elongation of *C. lanceolata* seedlings under different water extract of astringent seeds. HST represents the Hoagland nutrient solution treatment. AST represents the active aluminum stress treatment. LET and HET represent the low-concentration and high-concentration water extracts treatments under active aluminum stress, respectively. HS+LET and HS+HET indicate the low-concentration and high-concentration water extracts treatments under Hoagland nutrient solution, respectively.

## References

[B1] Aastveit A. H. Aastveit K. (1993). Effects of genotype-environment interactions on genetic correlations. Theor. Appl. Genet. 86, 1007–1013. doi: 10.1007/BF00211054 24194010

[B2] Arathi H. S. (2011). Selective embryo abortion in a perennial tree-legume: a case for maternal advantage of reduced seed number per fruit. J. Plant Res. 124, 675–681. doi: 10.1007/s10265-010-0400-z 21249418

[B3] Bai B. Bian H. Zeng Z. Hou N. Shi B. Wang J.. (2017). miR393-Mediated auxin signalingregulation is involved in root elongation inhibition in response to toxic aluminum stress in barley. Plant Cell Physiol. 58, 426–439. doi: 10.1093/pcp/pcw211 28064248

[B4] Berhe M. You J. Dossa K. Li D. Zhou R. Zhang Y.. (2024). Examining chlorophyll extraction methods in sesame genotypes: uncovering leaf coloration effects and anatomy variations. Plants-Basel 13, 1589. doi: 10.3390/plants13121589 38931021 PMC11207426

[B5] Burd M. (1998). ‘Excess’ flower production and selective fruit abortion: A model of potential benefits. Ecology 79, 2123–2123. doi: 10.2307/176715

[B6] Cárcamo M. P. Reyes-Díaz M. Rengel Z. Miren A. Rebeca P. O. G. Adriano N. N.. (2019). Aluminum stress differentially affects physiological performance and metabolic compounds in cultivars of highbush blueberry. Sci. Rep. 9, 11275. doi: 10.1038/s41598-019-47569-8 31375763 PMC6677737

[B7] Cardel Y. J. Koptur S. (2022). Locations of seed abortion in response to defoliation differ with pollen source in a native perennial legume herb. Am. J. Botany 109, 1730–1740. doi: 10.1002/ajb2.16055 36088615

[B8] Chen L. Wang S. (2003). Preliminary study of allelopathy of root exudates of Chinese fir. Acta Ecologica Sinica 23, 393–398. doi: 10.3321/j.issn:1000-0933.2003.02.025

[B9] Chen Y. Wu Y. H. Wu C. Lin S. (2020). Comparative analysis reveals the metabolic characteristics of astringent seeds of Chinese fir (*Cunninghamia lanceolata* (Lamb). Hook). during astringent compounds accumulation stages. Forests 11, 1206. doi: 10.3390/f11111206

[B10] Chen T. Xie M. Jiang Y. Yuan T. (2022). Abortion occurs during double fertilization and ovule development in *Paeonia ludlowii* . J. Plant Res. 135, 295–310. doi: 10.1007/s10265-021-01366-5 35059894 PMC8894304

[B11] Chomel M. Guittonny-L. M. Fernandez C. Gallet C. DesRochers A. Paré D.. (2016). Plant secondary metabolites: a key driver of litter decomposition and soil nutrient cycling. J. Ecol. 104, 1527–1541. doi: 10.1111/1365-2745.12644

[B12] Drabek O. Boruvka L. Mladkova L. Kočárek M. (2003). Possible method of aluminium speciation in forest soils. J. Inorganic Biochem. 97, 8–15. doi: 10.1016/S0162-0134(03).00259-9 14507455

[B13] Du B. Cao Y. Zhou J. Chen Y. Ye Z. Huang Y.. (2024). Sugar import mediated by sugar transporters and cell wall invertases for seed development in *Camellia oleifera* . Horticulture Res. 11, uhae133. doi: 10.1093/hr/uhae133 PMC11226869 38974190

[B14] Eticha D. Stass A. Horst W. J. (2005). Cell-wall pectin and its degree of methylation in the maize root-apex: significance for genotypic differences in aluminium resistance. Plant Cell Environment 28, 1410–1420. doi: 10.1111/j.1365-3040.2005.01375.x

[B15] Godoy L. J. Ferraz M. V. Silva L. D. Ferraz M. V. (2016). Accumulation and biomass partition and nutrients per tropical ornamental plants grown in Ribeira valley region. Ornamental Horticulture 22, 277. doi: 10.14295/oh.v22I3.933

[B16] Gordo S. G. Ruiz M. R. Palma J. M. Corpas F. J. (2024). Comparative analysis of catalase activity in plants: spectrophotometry and native PAGE approaches. Methods Mol. Biol. (Clifton N.J.) 2798, 213–221. doi: 10.1007/978-1-0716-3826-2-15 38587746

[B17] Jayakodi M. Madheswaran M. Adhimoolam K. Perumal S. Manickamet D. Kandasamy T.. (2019). Transcriptomes of Indian barnyard millet and barnyardgrass reveal putative genes involved in drought adaptation and micronutrient accumulation. Acta Physiologiae Plantarum. 41, 66. doi: 10.1007/s11738-019-2855-4

[B18] Jiang Y. Hu Z. Han Z. Zhang J. Han S. Lin H. (2022). Growth characteristics of *Cunninghamia lanceolata* in China. Sci. Rep. 12, 18179. doi: 10.1038/s41598-022-22809-6 36307492 PMC9616935

[B19] Kato-Noguchi H. Mizutani J. Hasegawa K. (1994). Allelopathy of oats. II. Allelochemical effect ofL-Tryptophan and its concentration in oat root exudates. J. Chem. Ecol. 20, 315–319. doi: 10.1007/BF02064440 24242057

[B20] Kochian L. V. Piñeros M. A. Liu J. Magalhaes J. V. (2015). Plant adaptation to acid soils: the molecular basis for crop aluminum resistance. Annu. Rev. Plant Biol. 66, 571–598. doi: 10.1146/annurev-arplant-043014-114822 25621514

[B21] Kumar N. Singh K. Giri A. Kumar A. Joshi A. Yadav S.. (2024). Physiological and molecular insights into the allelopathic effects on agroecosystems under changing environmental conditions. Physiol. Mol. Biol. Plants 30, 417–433. doi: 10.1007/s12298-024-01440-x 38633277 PMC11018569

[B22] Lee H. Ro N. Jeong H. Kwon J. Jo J. Ha Y.. (2016). Genetic diversity and population structure analysis to construct a core collection from a large germplasm. BMC Genet. 17, 142. doi: 10.1186/s12863-016-0452-8 27842492 PMC5109817

[B23] Li S. Geng. X. Chen. S. Liu K. Yu S. Wang X.. (2021). The co-expression of genes involved in seed coat and endosperm development promotes seed abortion in grapevine. Planta 254, 87–87. doi: 10.1007/s00425-021-03728-8 34585280

[B24] Li Z. Sun L. Cao Y. Ren J. Leng P. Hu H. (2009). Study on low frequency of ovule fertilization and high rate of seed abortion in ‘Mopanshi’ persimmon. Iv Int. Symposium Persimmon 833, 113–118. doi: 10.17660/actahortic.2009.833.17

[B25] Lin S. Chen Y. Wu C. Sun W. Li Z. Chen H.. (2022). Chinese fir genome and the evolution of gymnosperms. BioRxiv: preprint. 10 (25), 513437. doi: 10.1101/2022.10.25.513437

[B26] Lin X. Ma Z. He Z. Chen Y. Peng Y. Lin S. (2017). Optimization of ultrasonic-assisted extraction process for polyphenols from the seeds of Chinese fir. J. For. Environ. 37, 47–53. doi: 10.13324/j.cnki.jfcf.2017.01.008

[B27] Luo F. Chen A. Li F. Zhang L. Xie S. Zhang H.. (2014). Seasonal dynamics of soil active aluminum content in Chinese fir plantations of different ages in acid rain area. J. Fujian Agric. Forestry Univ. (Natural Sci. Edition) 43, 470–477. doi: 10.3969/j.issn.1001-389X.2014.02.007

[B28] Ma Z. Lin S. (2019). Transcriptomic Revelation of phenolic compounds involved in aluminum toxicity responses in roots of *Cunninghamia lanceolata* (Lamb.). Hook. Genes 10, 835. doi: 10.3390/genes10110835 31652726 PMC6896160

[B29] Meyer K. M. Soldaat L. L. Auge H. Thulke H. H. (2014). Adaptive and selective seed abortion reveals complex conditional decision making in plants. Am. Naturalist 183, 376–383. doi: 10.1086/675063 24561600

[B30] Mika A. Lüthje S. (2003). Properties of guaiacol peroxidase activities isolated from corn root plasma membranes. Plant Physiol. 132, 1489–1498. doi: 10.1104/pp.103.020396 12857829 PMC167087

[B31] Morales M. Munné-Bosch S. (2019). Malondialdehyde:facts and artifacts. Plant Physiol. 180, 1246–1250. doi: 10.1104/pp.19.00405 31253746 PMC6752910

[B32] Muhammad R. Yan L. Wu X. Hussain S. Aziz O. Muhammad I.. (2018). Boron reduces aluminum-induced growth inhibition, oxidative damage and alterations in the cell wall components in the roots of trifoliate orange. Ecotoxicology Environ. Safety 153, 107–115. doi: 10.1016/j.ecoenv.2018.02.002 29425841

[B33] Nguyen Q. T. Kisiala A. Andreas P. Neil Emery R. J. Narine S. (2016). Soybean seed development: fatty acid and phytohormone metabolism and their interactions. Curr. Genomics 17, 241–260. doi: 10.2174/1389202917666160202220238 27252591 PMC4869011

[B34] Orgogozo V. Morizot B. Martin A. (2015). The differential view of genotype-phenotype relationships. Front. Genet. 6. doi: 10.3389/fgene.2015.00179 PMC4437230 26042146

[B35] Ofoe R. Thomas R. Asiedu S. Wang-Pruski G. Fofana B. Abbey L. (2022). Aluminum in plant: Benefits, toxicity and tolerance mechanisms. Front. Plant Sci. 13, 1085998. doi: 10.3389/fpls.2022.1085998 36714730 PMC9880555

[B36] Parkash A. O. Dhinu Y. Nisha W. Kashyap P. L. Sharma P. Tiwari R. (2024). Root exudates and their significance in abiotic stress amelioration in plants: a review. J. Plant Growth Regulation 43, 1736–1761. doi: 10.1007/s00344-024-11237-7

[B37] Plomin R. Asbury K. (2005). Nature and nurture: genetic and environmental influences on behavior. Ann. Am. Acad. Polit Ss 600, 86–98. doi: 10.1177/0002716205277184

[B38] Sade H. Meriga B. Surapu V. Gadi J. Sunita M. S. Suravajhala P.. (2016). Toxicity and tolerance of aluminum in plants: tailoring plants to suit to acid soils. Biometals 29, 187–210. doi: 10.1007/s10534-016-9910-z 26796895

[B39] Schroeter S. A. Eveillard D. Chaffron S. Zoppi J. Kampe B. Lohmann P.. (2022). Microbial community functioning during plant litter decomposition. Sci. Rep. 12, 7451. doi: 10.1038/s41598-022-11485-1 35523988 PMC9076648

[B40] Shen S. Liang X. Zhang L. Zhao X. Liu Y. Lin S.. (2020). Intervening in sibling competition for assimilates by controlled pollination prevents seed abortion under postpollination drought in maize. Plant Cell Environment 43, 903–919. doi: 10.1111/pce.13704 31851373

[B41] Shen Y. Zhang Z. Xue Y. (2021). Study on the new dynamics and driving factors of soil pH in the red soil, hilly region of South China. Environ. Monit Assess 193, 304. doi: 10.1007/s10661-021-09080-4 33900476

[B42] Shetty R. Vidya C. S. Prakash N. B. Lux A. Vaculík M. (2020). Aluminum toxicity in plants and its possible mitigation in acid soils by biochar: a review. Sci. Total Environment 765, 142744. doi: 10.1016/j.scitotenv.2020.142744 33092837

[B43] Soln K. Klemencic M. Koce D. (2022). Plant cell responses to allelopathy: from oxidative stress to programmed cell death. Protoplasma 259, 1111–1124. doi: 10.1007/s00709-021-01729-8 34993622

[B44] Sun L. Zhang M. Liu X. Mao Q. Shi C. Kochian L. V.. (2020). Aluminium is essential for root growth and development of tea plants (*Camellia sinensis*). J. Integr. Plant Biol. 62, 984–997. doi: 10.1111/jipb.12942 32320136 PMC7383589

[B45] Venzhik Y. V. Shchyogolev S. Y. Dykman L. A. (2019). Ultrastructural reorganization of chloroplasts during plant adaptation to abiotic stress factors. Russ J. Plant Physiol. 66, 850–863. doi: 10.1134/S102144371906013X

[B46] Vicente V. P. Carlos D. O. Aurelio G. C. Rosa María P. C. (2020). Root exudates: from plant to rhizosphere and beyond. Plant Cell Rep. 39, 3–17. doi: 10.1007/s00299-019-02447-5 31346716

[B47] Wang X. Chen J. Hu L. Zhang J. Xiao F. Zhang S.. (2023). Embryological observations on seed abortion in *Hibiscus Syriacus* L. and physiological studies on nutrients, enzyme activity and endogenous hormones. BMC Plant Biol. 23, 665. doi: 10.1186/s12870-023-04669-y 38129795 PMC10740302

[B48] Wei Y. Jiang C. Han R. Xie Y. Liu L. Yu Y. (2020). Plasma membrane proteomic analysis by TMT-PRM provides insight into mechanisms of aluminum resistance in tamba black soybean roots tips. PeerJ 8, e9312. doi: 10.7717/peerj.9312 32566407 PMC7293186

[B49] Xie D. Ma X. Mohammad Z. R. Yang M. Huang X. Li J.. (2019). Thermo-sensitive sterility and self-sterility underlie the partial seed abortion phenotype of *Litchi chinensis* . Sci. Hortic-Amsterdam 247, 156–164. doi: 10.1016/j.scienta.2018.11.083

[B50] Yan L. Muhammad R. Li S. Cheng J. Jiang C. (2023). Harnessing the power of exogenous factors to enhance plant resistance to aluminum toxicity; a critical review. Plant Physiol. Biochem. 203, 108064. doi: 10.1016/j.plaphy.2023.108064 37783071

[B51] Yang J. Fan W. Zheng S. (2019). Mechanisms and regulation of aluminum-induced secretion of organic acid anions from plant roots. J. Zhejiang Univ. Sci. B. 20, 513–527. doi: 10.1631/jzus.B1900188 31090277 PMC6568218

[B52] Yang L. Qi Y. Jiang H. Chen L. (2013). Roles of organic acid anion secretion in aluminium tolerance of higher plants. BioMed. Res. Int. 2013, 173682. doi: 10.1155/2013/173682 23509687 PMC3591170

[B53] Yang X. Wang X. Xiao S. Liu Z. Zhou X. Du G.. (2021). Dominant plants affect litter decomposition mainly through modifications of the soil microbial community. Soil Biol. Biochem. 161, 108399. doi: 10.1016/j.soilbio.2021.108399

[B54] Zahir S. Zhang F. Chen J. Zhu S. (2021). Determination of oxidative stress and antioxidant enzyme activity for physiological phenotyping during heavy metal exposure. Methods Mol. Biol. 2326, 241–249. doi: 10.1007/978-1-0716-1514-0-17 34097273

[B55] Zhang M. Steele M. A. Yi X. (2013). Reconsidering the effects of tannin on seed dispersal by rodents: evidence from enclosure and field experiments with artificial seeds. Behav. Process 100, 200–207. doi: 10.1016/j.beproc.2013.09.010 24161819

[B56] Zhang J. Yan X. Dayananda B. Cheng J. Luo Y. (2024). Frequency-dependent seed selection by rodents: response to seed tannins and sizes. Glob Ecol. Conserv. 54, e03073. doi: 10.1016/j.gecco.2024.e03073

[B57] Zheng S. (2010). Crop production on acidic soils: overcoming aluminium toxicity and phosphorus deficiency. Ann. Botany 106, 183–184. doi: 10.1093/aob/mcq134 20570831 PMC2889811

[B58] Zheng A. Li C. (2004). Influences and improvement of Al toxin on plants in acid red soil. Hubei Agricultural Sci. 6, 41–43. doi: 10.3969/j.issn.0439-8114.2004.06.014

[B59] Zheng P. Shen M. Liu R. Cai X. Lin J. Wang L.. (2023). Revealing further insights into astringent seeds of Chinese fir by integrated metabolomic and lipidomic analyses. Int. J. Mol. Sci. 24, 15103. doi: 10.3390/ijms242015103 37894783 PMC10607028

[B60] Zhou N. Liu P. Wang Z. Xu G. (2011). The effects of rapeseed root exudates on the forms of aluminum in aluminum stressed rhizosphere soil. Crop Prot. 30, 631–636. doi: 10.1016/j.cropro.2011.02.011

