# Supplementary figures and images for: A potential role of a special type of abortive seeds in Cunninghamia lanceolata: promoting the growth of healthy seedlings in active aluminum ions-rich soil
Source: Front Plant Sci. 2024 Nov 8;15:1482355. doi: 10.3389/fpls.2024.1482355 (PMC11581864; doi:10.3389/fpls.2024.1482355)

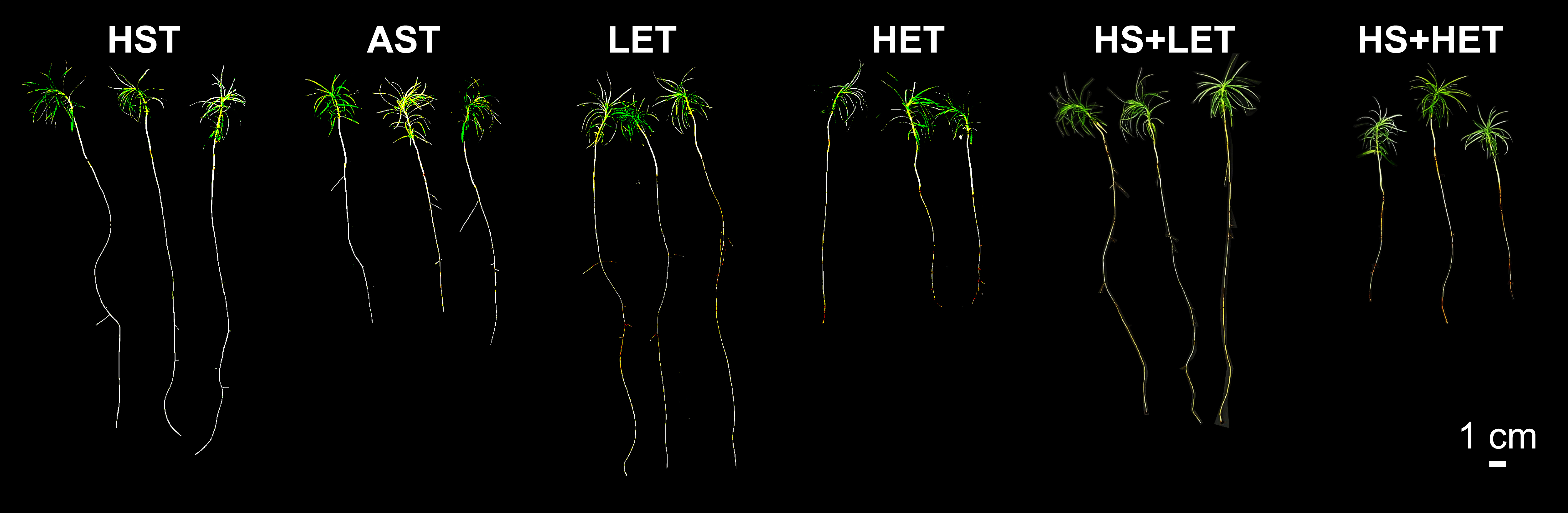

Supplement: Supplementary Figure 1 — Differences in root elongation of C. lanceolata seedlings under different water extract of astringent seeds. HST represents the Hoagland nutrient solution treatment. AST represents the active aluminum stress treatment. LET and HET represent the low-concentration and high-concentration water extracts treatments under active aluminum stress, respectively. HS+LET and HS+HET indicate the low-concentration and high-concentration water extracts treatments under Hoagland nutrient solution, respectively. [file Image1.jpeg]
